# Mycoplasma pneumoniae Associated Acute Transverse Myelitis: An Atypical Clinical Presentation in an Adolescent Child

**DOI:** 10.7759/cureus.17259

**Published:** 2021-08-17

**Authors:** Chong Bin He, Madelyn Kahana

**Affiliations:** 1 Pediatrics, University of Central Florida College of Medicine, Orlando, USA; 2 Pediatric Critical Care Medicine, Nemours Children's Hospital, Orlando, USA

**Keywords:** mycoplasma pneumoniae, acute transverse myelitis, immune-mediated inflammatory disorder, central nervous system disorder, neurologic disorder

## Abstract

We report an atypical case of a 15-year-old pediatric patient diagnosed with Mycoplasma pneumoniae* *associated acute transverse myelitis (ATM). The patient had no prodromal or pulmonary symptoms that are commonly associated with mycoplasma infection. Yet, the patient exhibited acute bilateral lower extremity paralysis, paresthesia, decreased sensation at the level of T11 and below, bowel and bladder dysfunction, and thrombocytopenia. Magnetic resonance imaging of the spinal cord revealed transverse myelitis from T10 to the end of the conus medullaris. The patient showed only slow clinical improvement despite therapy consisting of azithromycin, high-dose intravenous methylprednisolone, intravenous immunoglobulin, and plasmapheresis. This report calls attention to the importance of early identification of mycoplasma as an underlying cause of ATM and the potential consequences of delayed detection and treatment: more severe neurologic complications, prolonged hospitalization, and unfavorable clinical outcomes.

## Introduction

Acute transverse myelitis (ATM) is a rare acquired immune-mediated inflammatory disorder of the spinal cord and the central nervous system. It is characterized by a sudden onset of back pain followed by loss of sensory, motor, or autonomic function in the form of sphincter disturbances. Neurologic symptoms of ATM can develop rapidly over hours or sub-acutely over weeks. The most common first sign is the change in the patient's sensation. Motor and sensory changes are typically bilateral. Motor dysfunction in ATM involves flaccid motor weakness and hyporeflexia. Sensory changes consist of paresthesia, hyperesthesia, or numbness in a band-like transverse distribution at definable dermatomal levels. Bowel or bladder dysfunction can also be seen. The incidence of ATM in pediatrics is low, with two per every million children per year. However, about 20% of all pediatric acquired demyelinating syndrome cases are associated with ATM. The age distribution of ATM is bimodal, with children under five and those older than ten years of age making up the primary groups [1-3]. Studies have found that 66-85% of pediatric patients with ATM develop longitudinally extensive transverse myelitis (LETM) and present with spinal cord damages in more than three vertebral segments. Overall, the prognosis of ATM is better in children compared to adult patients [2]. 

ATM is caused by a mixed inflammatory process that affects the grey and white matter, including the neurons, axons, oligodendrocytes, and myelin [4]. The causes of ATM are numerous: from bacterial, viral, autoimmune, post-vaccination to being part of the neoplastic or paraneoplastic processes [5]. Thus, ATM could manifest as a monophasic demyelinating disorder, the initial presentation of a chronic, relapsing demyelinating syndrome, or a neurologic sequela of a systemic autoimmune condition. Around 80% of ATM cases in children belong to the monophasic idiopathic category, and those patients tend to experience an antecedent respiratory or gastrointestinal infection. Furthermore, patients with monophasic idiopathic ATM experience a faster progression of symptoms [2,3,5]. 

Mycoplasma pneumoniae is a common cause of respiratory infections. However, it is not uncommon for patients who have contracted M. pneumoniae to experience extrapulmonary symptoms. It has been reported that M. pneumoniae can affect almost every organ system. Neurological complications from this bacterium are found in 0.01% to 4.8% of the patients [6]. Symptoms of M. pneumoniae­ associated ATM typically arise two to four weeks after a prodromal respiratory infection and involves the thoracic spinal cord. Due to this neurologic complication's rarity, the exact mechanism of how M. pneumoniae­ affects the central nervous system, particularly the spinal cord, is unclear. There are many theories regarding how M. pneumoniae­ infection leads to transverse myelitis. It is proposed that the inflammatory processes that occur in the grey and white matter of the spinal cord could be caused by the direct bacterial invasion or by a post-infectious immune response. The post-infectious immune response is due to the activating effect of M. pneumoniae­ on B-cells or molecular mimicry between M. pneumoniae­ and myelin sheath proteins that lead to the production of anti-neuronal antibodies [2,6].

Correctly diagnosing M. pneumoniae­ associated ATM can be rather challenging as ATM can be caused by various pathology. In most cases, the cause of ATM is identified through elimination after MRI or lumbar puncture findings reveal inflammation of the spinal cord. Evaluation involves differentiating the various causes of myelitis, including CNS inflammatory demyelinating disorder, systemic inflammatory and autoimmune disorders, infections, paraneoplastic syndromes, and cancers. When diagnosing M. pneumoniae­ associated ATM, first, it is necessary to verify whether the patient has an active or recent infection of M. pneumoniae­. Recent mycoplasma infection can be confirmed through M. pneumoniae­ serum serology that shows Mycoplasma IgM or IgG titers. An increasing IgM level indicates an active infection. It has also been shown that a throat-swab PCR is sensitive for identifying acute M. pneumoniae­ infection. Successfully isolating M. pneumoniae­ from a patient's cerebrospinal fluid is uncommon [5,6]. It is important to note that M. pneumoniae­ associated ATM is often only diagnosed after all other causes and other infectious causes have been eliminated. 

There is no definitive treatment for M. pneumoniae­ associated ATM or ATM from other causes. The current first-line treatment is high-dose corticosteroids for three to five days followed by an oral taper for three to four weeks [2,7,8]. Second-line therapy is plasmapheresis if the patient shows no improvement after steroid treatment. Salloum et al. have reported that plasmapheresis after steroids is particularly beneficial for patients with LETM. Intravenous immunoglobulin (IVIG) administration has also been reported as an alternative for treating ATM after steroids. While antimicrobial treatment is controversial, using macrolides in ATM is thought to be beneficial due to their effects on suppressing inflammatory cytokines in addition to potentially treating the active infection of M. pneumoniae­. Furthermore, timely and aggressive rehabilitation and physical therapy are the mainstays in managing ATM patients [2]. 

Most monophasic idiopathic ATM patients show at least a partial recovery one to three months after the onset of symptoms. Many reports have shown that about 50% of pediatric patients have a full recovery within two years [2,8]. Favorable prognostic factors in ATM patients include plateauing of neurologic symptoms in less than eight days of onset, showing signs of recovery within one week of symptom onset, walking independently in less than one month, and less than ten years. Unfavorable outcomes of ATM are associated with rapid deterioration of neurologic symptoms in less than 24 hours, complete paraplegia, supraspinal symptoms, severe motor weakness, sphincter involvement, and spinal atrophy on MRI [1,8,9].

## Case presentation

A 15-year-old white male patient with Asperger's syndrome, anxiety disorder, and attention deficit hyperactivity disorder presented with acute onset of bilateral lower extremity weakness, paresthesia, and decreased sensation. One day before admission, the patient experienced paresthesia in his bilateral thigh and calf muscles but had a normal movement of the lower extremities. On admission, the patient could not move his legs normally, and numbness had progressed down his entire legs bilaterally. He had difficulty sitting and bending forward due to back pain. He also had no spontaneous urination or bowel movement since the onset of weakness. The patient had no history of similar symptoms or problems with urination and defecation. He denied symptoms in the upper extremities, fever, flu-like symptoms, urinary tract infections, and other infections. He had no pets, tick bits, recent travel, or recent vaccinations. The patient had normal development and had no age-related gross motor milestone delays. He has been receiving social and behavioral therapies for his Asperger's syndrome. His medications included amphetamine-dextroamphetamine, sertraline, and risperidone.

The patient's review of the system was positive for abdominal pain and constipation, lower extremity weakness, trouble with walking, lower back pain, and urinary retention. The neurologic system was positive for bilateral leg weakness, numbness, decreased sensation, and negative for headache, slurred speech, facial weakness, hearing loss, dysphagia, irritability, and change in behaviors. On physical examination, the patient was alert, interactive, and not in distress. He was afebrile and had normal blood pressure and heart rate. His oxygen saturation was above 95% on room air. Cardiovascular and pulmonary exams were unremarkable. There were active bowel sounds and no abdominal guarding. His bladder was full and palpable. The patient was oriented to person, place, and time and his cranial nerves were grossly intact. However, there was a flaccid tone in the lower extremities and 0/5 strength in hips, knees, feet, and toes. The rectal tone was absent. Reflexes in the bilateral knees, ankles and plantar reflexes were absent. Upper extremities were unaffected with normal tone, strength, and reflexes. The patient had decreased sensation to fine touch, pinprick test, and temperature in the bilateral lower extremities at the sensory level of T11-12 and below.

**Figure 1:**
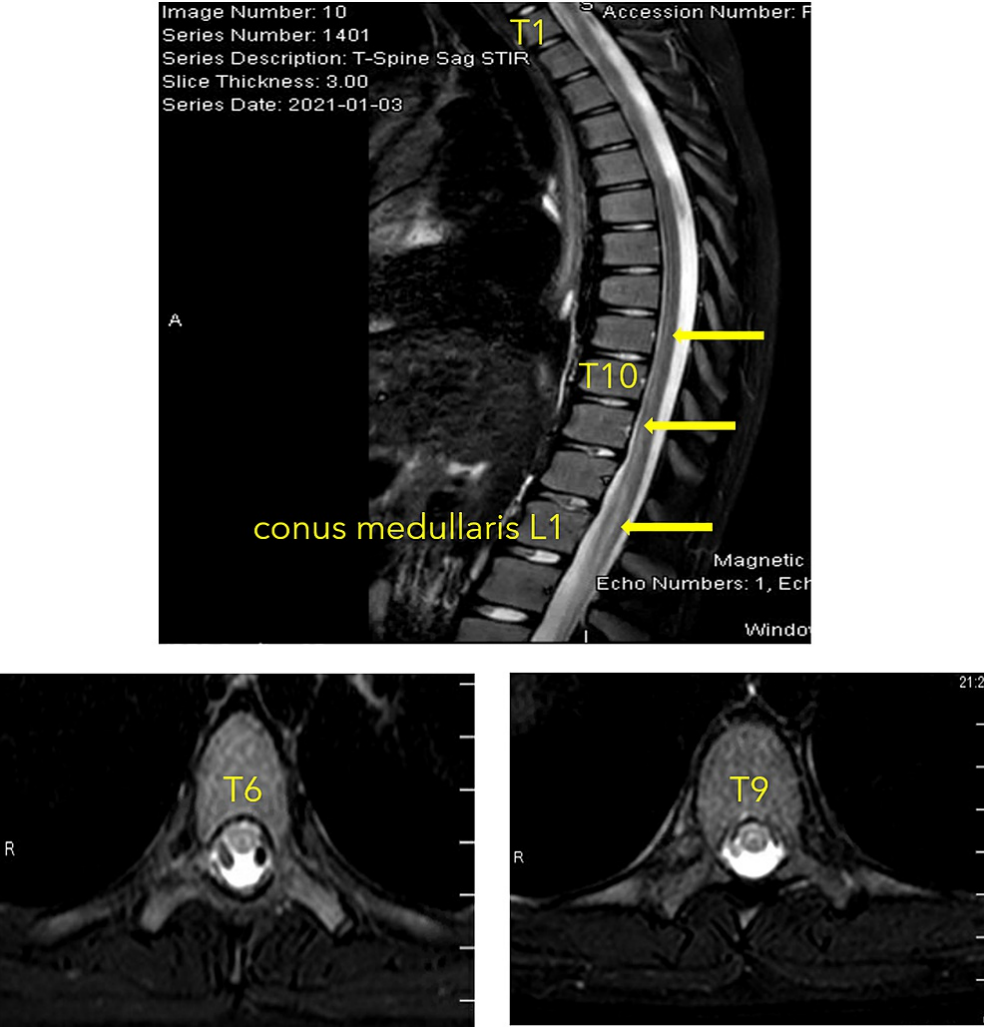
Magnetic Resonance Imaging of the Thoracic Spine There are abnormal T2/STIR signals (arrows) involving the distal spinal cord at the level of the lower thoracic spine beginning at approximately T8-T9 and extending inferiorly to the level of the conus medullaris.

**Figure 2:**
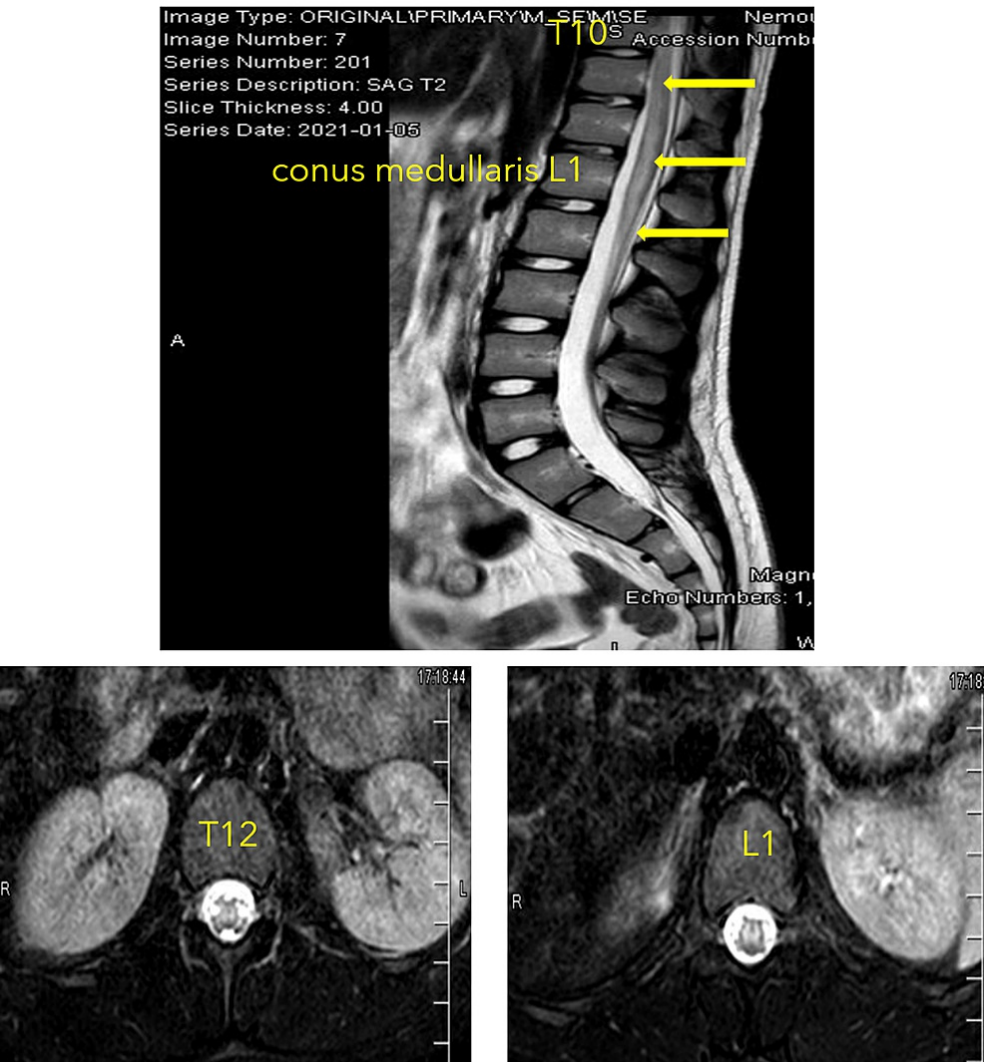
Magnetic Resonance Imaging of the Lumbar Spine There are abnormal T2/STIR signals (arrows) that extend from T10 to the level of conus medullaris. The high-intensity abnormal signals appear to terminate at L2.

Initial workup in the emergency room included lab work and imaging studies. Complete blood count revealed thrombocytopenia with a platelet count of 75 x10^3^/μL (ref: 150-400x10^3^/μL) but otherwise normal hemoglobin (14.7 g/dL) (ref: 11.0-14.3 g/dL), hematocrit (43%) (ref: 31.4-41.0%), and white blood cell counts (5.5 x10^3^/μL) (ref: 5.24-9.74x10^3^/μL). Blood coagulation, complete metabolic panel, erythrocyte sedimentation rate, and C-reactive protein values were unremarkable. Urinalysis and drug screen were only positive for amphetamine. The respiratory panels tested for common viral, COVID-19, and bacterial respiratory infections were negative. A chest radiograph showed clear lung fields. The brain's magnetic resonance imaging showed grossly normal structure with no evidence of multiple sclerosis or acute disseminated encephalomyelitis. Magnetic resonance imaging of the cervical, thoracic, and lumbar spine was also performed. Cervical spine imaging was unremarkable. However, the thoracic and lumbar spine images showed significant abnormal high T2 signals extended from T10 to the end of the conus medullaris (Figure 1). The abnormal signals appear circumferentially in the central portion of the cord with sparing of the peripheral. Furthermore, the images also revealed mild thickening of the conus medullaris (Figure 2). Based on the magnetic resonance imaging, the patient's provisional diagnosis was transverse myelitis. 

Since the etiology of the patient's weakness and sensory loss was still unclear, and the patient was still at risk of developing cardiovascular instability and respiratory failure, the patient was transferred to the pediatric intensive care unit for continuous cardiorespiratory monitoring and diagnostic workup. In the intensive care unit, the patient received a platelet transfusion. A lumbar puncture was then performed to identify the etiology of inflammatory and infectious causes. Furthermore, transverse myelitis lab work was ordered, including the common bacterial and viral panel, CNS inflammatory demyelinating disorder markers, systemic inflammatory disorder markers, and paraneoplastic markers. Lastly, lab studies for copper, vitamin B12, and vitamin E were ordered to rule out myelopathy due to vitamin or mineral deficiency.

CSF fluid collected after the lumbar puncture procedure was sterile and colorless with 2 WBC/uL (ref: <100/μL) and 4 RBC/uL (ref: 0-5/μL). There was a normal CSF glucose concentration (56mg/dL) (ref: 41-84mg/dL) and an elevated protein concentration (68mg/dL) (ref: <48mg/dL). IgG index of the CSF was not obtained. Most lab studies, including an infectious panel, CNS autoimmune markers, systemic autoimmune markers, paraneoplastic markers, and vitamin/mineral, were all unremarkable. The detailed list of the workup can be reviewed in Table 1 and 2. The positive findings included an elevated M. pneumoniae­ serum IgM (1630 U/ml) (ref: 0-769U/ml) and IgG (660 U/ml) (ref: 0-99 U/ml) and a high myelin basic protein level in the CSF (7.8ng/mL) (ref: 0-3.8ng/mL).

**Table 1:** Positive Laboratory Findings

Categories	Laboratory Studies
Infectious	Mycoplasma IgM and IgG
CNS/CNS Autoimmune	Myelin basic protein, CSF: high protein (normal glucose, low RBC, and WBC)

**Table 2:** Negative Laboratory Findings CSF- Cerebrospinal fluid; CMV- Cytomegalovirus; EBV- Epstein-Barr virus; HTLV- human T-lymphotropic virus; MOG- Myelin oligodendrocyte glycoprotein; NMO/AQP4- Neuromyelitis optica /Aquaporin-4

Categories	Laboratory Studies
Infectious	The respiratory panel, CSF meningitis panel, CMV, EBV, HTLV 1 & 2, Lyme disease, M. Pneumoniae, West Nile, HIV
CNS/ CNS Autoimmune	Oligoclonal band, Autoimmune encephalitis panel, MOG antibody, NMO/AQP4 antibody, Paraneoplastic antibody
Systemic Autoimmune	Angiotensin-1 converting enzyme, dsDNA, Rh factor, Cyclic citrullinated peptide, IgA, IgM, IgG levels
Vitamins/Minerals	Ceruloplasmin, Copper, Vitamin B12, Vitamin E

**Figure 3:**
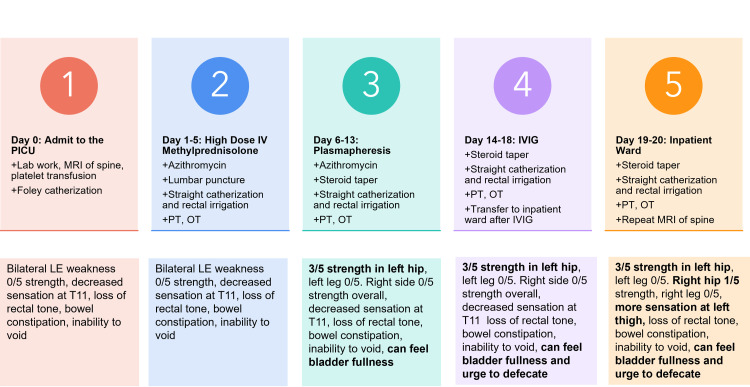
Summary of Patient's Hospital Course PICU- Pediatric Intensive Care Unit; PT/OT- Occupational/Physical Therapy; Bilateral LE- Bilateral Lower Extremity

Figure 3 summarizes the timeline and the management of the patient during his hospitalization. With the provisional diagnosis of transverse myelitis by magnetic resonance imaging, the patient was treated with intravenous high-dose methylprednisolone daily on hospital day one for five days. The patient also required regular straight catheterization and rectal irrigation. Physical and occupational therapy were consulted to assist with the patient's recovery. Azithromycin was added to his medical regimen on hospital day four because of the positive M. pneumoniae­ serum titers for a total of five days. Throughout the five-day high-dose glucocorticoid treatment, the patient's neurologic exam remained unchanged compared to admission. He continued to experience bilateral lower extremity weakness with 0/5 strength, decreased sensation at the level of T11, loss of rectal tone, bowel constipation, and inability to void. It was important to note that the patient did not experience further motor and sensory loss. Because the patient experienced little improvement, plasma exchange was initiated for five courses every other day while on a scheduled steroid taper. After the third course of plasma exchange, the patient demonstrated 3/5 strength in left hip flexors, extensors, abductors, and adductors. However, the patient's strength remained 0/5 for the rest of the left and the entire right leg. The patient could sense bladder fullness but was unable to urinate. By the time plasma exchange was completed, the patient could also feel the urge to defecate. Intravenous immunoglobulin (IVIG) was initiated on hospital day 14 due to the slow recovery of the patient's motor and sensory functions. 

After completing IVIG, the patient was transferred to the inpatient ward for transition to inpatient rehabilitation since he had demonstrated cardiorespiratory stability in the intensive care unit. At that time, the patient's right hip showed 1/5 strength in hip flexors, extensors, abductors, and adductors. The patient also regained more sensation on the left thigh. Both M. pneumoniae­ serum IgM (<770 U/ml) (ref: 0-769U/ml) and IgG (110 U/ml) (ref: 0-99 U/ml) were lower than the previous measurement. Repeat MRI of the thoracic and lumbar spine revealed improvement in abnormal T2 hyperintense signals in the region from T8-T9 through T11 levels. However, there was an increased enhancement of the conus medullaris and nerve roots of the cauda equina. The patient's diagnosis remained transverse myelitis associated with M. pneumoniae infection. However, since the patient's slow improvement in motor, sensory, and autonomic function, atypical initial presentation, and increased enhancement in conus medullaris and cauda equina in repeat MRI, the patient continued to be followed by rheumatology and neurology. 

## Discussion

Acute transverse myelitis (ATM) is an acquired neuro-immune spinal disorder characterized by rapid motor, sensory, and autonomic dysfunction. ATM is triggered by many causes, including molecular mimicry after an infection, post-vaccination, recurrent autoimmune conditions of the central nervous system (CNS), active bacterial or viral infection, and part of the neoplastic/paraneoplastic syndrome. Furthermore, at times, ATM occurs without any positive diagnostic test. Moreover, ATM can be mistaken for other diseases involving the CNS, such as vascular myelopathies, metabolic or nutritional myelopathies, neoplasm, acute flaccid myelitis, and acute inflammatory demyelinating polyneuropathy. ATM can be differentiated from other neurological diseases in three aspects. First, patients with ATM typically present with sensory, motor, and autonomic dysfunction attributed to the spinal cord and progress to nadir between four hours and 21 days. Their motor and sensory dysfunction tend to manifest bilaterally. Furthermore, the sensory loss can be clearly located starting at one specific dermatome. Second, the patient's CSF tends to demonstrate increased cell count and higher IgG index. Third, spinal MRI of the patient typically shows T2 signal changes and the absence of compression cord lesion [10]. 

Here we report a 15-year-old white male patient in his usual state of health presented with acute bilateral leg weakness and numbness, decreased sensation at the level of T11 and below, loss of sphincter tone, and reduced urine output. He was diagnosed with ATM secondary to M. pneumoniae infection. His course was notable for a slow improvement in motor, sensory, and autonomic functions and worsening T2 signaling on spinal MRI despite antibiotics, steroids, plasma exchange, and IVIG treatments.

Our patient's clinical course was somewhat atypical. Theroux et al. have reported that most cases of pediatric ATM are idiopathic. Typically, the patient will have an antecedent respiratory or gastrointestinal infection or immunization before symptom presentation of ATM [3]. However, our patient did not exhibit any recent prodromal manifestations. It was also noted that the patient had normal white blood cell count and C-reactive and ESR levels. Our patient's blood work was significant for an isolated low platelet count. The presentations of thrombocytopenia, motor, sensory, and autonomic dysfunction could be due to various factors. The differential included post-viral infection, autoimmune disorders, active viral infections, and vitamin deficiency. However, most of the patient's infectious, CNS autoimmune, systemic autoimmune, paraneoplastic, and vitamin studies were unremarkable, except for elevation of myelin basic protein and high protein in CSF and presence of IgM and IgG for Mycoplasma pneumoniae. The reports of Gouveia et al. and Okoli et al. have provided a possible explanation of the patient's initial presentation. These two groups reported that immune thrombocytopenia could be observed during mycoplasma infections, although it is very uncommon [11,12].

Another perplexing aspect of this patient case was the delayed clinical improvement after the administration of antibiotics targeting the patient's positive mycoplasma serology in addition to high-dose steroids, plasmapheresis, and IVIG. In the case presented by MacFarlane et al., a 14-year-old girl was treated with erythromycin and prednisolone after diagnosing with mycoplasma associated ATM. The female patient showed regression of sensory loss in 48 hours, gain of leg power in five days, return of spontaneous micturition function in three weeks, and progression to walking with help in four weeks. The patient had a reduction in serum mycoplasma titer starting on day nine; mycoplasma titer was undetectable on day 23 in the hospital. Salloum et al. highlighted a 13-year-old male patient diagnosed with mycoplasma associated ATM and was treated with doxycycline, high dose methylprednisolone, and subsequently plasmapheresis due to lack of improvement on steroids. In two weeks, the patient showed improved sensory and motor functions and minimal neurological deficits in his two-month follow-up. Our patient's disease progression and response to treatment were worth noting since most case studies in the literature with mycoplasma associated ATM have shown significant neurological recovery after antibiotics and immunosuppressive therapies such as steroids, plasmapheresis, and IVIG [2,5,6,13]. 

There are several possible reasons for delayed clinical improvement in our patients with mycoplasma associated ATM. First, it is unclear whether the patient still had an active mycoplasma infection when presented to the hospital. He had no prodromal symptoms and signs of active mycoplasma infection. His elevated M. pneumoniae serology only indicated that he was exposed to this bacterium. If the patient had an active mycoplasma infection, it is arguable whether the antibiotics the patient received effectively treated his neurological symptoms. Most antibiotics are only effective in treating pulmonary complications due to mycoplasma. The reason is that neurological manifestation is primarily due to immunologic processes rather than direct invasion or assault of the CNS by M. pneumoniae. Second, the patient's improvement of mycoplasma serology after completing an entire course of azithromycin could be an indirect result of plasmapheresis rather than an indicator for the clearance of mycoplasma infection. Therefore, there could be a discrepancy between the mycoplasma serology level and the actual clinical presentation. Third, suppose the patient's neurological symptoms are due to an immunological mechanism. In that case, the lack of response to immunosuppressive therapies might be due to the extensive CNS destruction by the patient's immune processes that could not be picked up in the initial imaging study. As a result, the effect of immunosuppressive therapies lagged behind the immune destruction of the CNS. This idea is supported by the fact that the thoracic regions, which were heavily affected, showed improvement, but more lesions emerged in the conus medullaris and cauda equina.

Reports have shown that around 50% of patients with idiopathic ATM recover fully within two years. However, our patient's presentation met several negative prognostic factors, including rapid deterioration of neurologic symptoms in less than 24 hours, severe motor weakness, and sphincter involvement. Even though the exact mechanism that caused the patient's neurological symptoms was unclear, it was fortunate that our patient started physical and occupational therapies early in his hospital course. Aggressive rehabilitation and physical therapy are the keys to motor function recovery in ATM patients [2]. Due to his symptoms' slow improvement, the patient needs to be closely followed by neurology, rheumatology, physical therapy, and occupational therapy.

## Conclusions

This report describes an atypical presentation of M. pneumoniae infection and highlights the clinical presentation of ATM, a rare, complicated extrapulmonary manifestation of mycoplasma infection. As shown in our patient, practitioners need to be aware that not every pediatric patient who contracted M. pneumoniae would experience chest pain, cough, and mild flu-like symptoms such as chills and fever. Furthermore, we should not omit the severe extrapulmonary sequelae such as neurologic, cardiac, hepatic, and hematologic diseases associated with mycoplasma infection's active or recovery phase. Sometimes, as seen in our patients, the extrapulmonary manifestation could be the only symptom of mycoplasma infection. The extrapulmonary symptoms could be from a direct bacterial invasion of the organ or an immune-mediated process. Understanding the disease process is the key to initiating the correct treatment and stopping the disease's progression. When encountering pediatric patients with symptoms of ATM, it is also critical for practitioners to consider M. pneumoniae as the potential cause. This patient's case emphasizes the importance of early detection and treatment of M. pneumoniae associated ATM. It is important to highlight that delaying detection of the disease process could lead to more severe neurologic complications, prolonged hospitalization, more aggressive immunosuppressive treatment, and unfavorable clinical outcomes.
