# Correction: Mycoplasma pneumoniae Associated Acute Transverse Myelitis: An Atypical Clinical Presentation in an Adolescent Child

**DOI:** 10.7759/cureus.c51

**Published:** 2021-11-08

**Authors:** Chong Bin He, Madelyn Kahana

**Affiliations:** 1 Pediatrics, University of Central Florida College of Medicine, Orlando, USA; 2 Pediatric Critical Care Medicine, Nemours Children's Hospital, Orlando, USA

Dr. James Lee, Emergency Medicine Resident Physician (UCF College of Medicine HCA Healthcare GME Consortium, North Florida Regional Medical Center) has been removed from the author list of this article as HCA Healthcare’s external release processes were not followed. As a result, Dr. Lee and HCA Healthcare have requested that Dr. Lee be removed from the author list of this article. The corresponding author, Chong Bin He (Pediatrics, University of Central Florida College of Medicine, Orlando, FL USA), appropriately followed protocols for patient privacy at the facility where the patient was treated.

